# Association of domain-specific physical activity with albuminuria among prediabetes and diabetes: a large cross-sectional study

**DOI:** 10.1186/s12967-024-05061-6

**Published:** 2024-03-08

**Authors:** Bingquan Xiong, Yufan Wang, Juan He, Lisha Wang, Rui He, Min Zhu, Jiaxing Wang, Yingrui Li, Bin Liu, Kaihu Xiao, Qiang She

**Affiliations:** 1 Department of Cardiology, The Second Affiliated Hospital of Chongqing Medical University, No.74, Linjiang Road, Chonqing, 400010 China; 2 Department of Cardiovascular Medicine Intensive Care Unit, The Second Affiliated Hospital of Hainan Medical University, Hainan, China; 3 Department of Respiratory and Critical Care Medicine, The Second Affiliated Hospital of Chongqing Medical University, No.74, Linjiang Road, Chonqing, 400010 China; 4 Department of Geriatrics, The First People’s Hospital of Neijiang, No. 41 Tuozhong Lane, Jiaotong Road, Neijiang, 641000 Sichuan China; 5 Department of Endocrinology and Metabolism, The First Affiliated Hospital of Jinan University, No. 613, Huang Pu Avenue West, Guangzhou, Guangdong China

**Keywords:** Physical activity, Diabetes, Albuminuria, Risk, Lifestyle

## Abstract

**Background:**

Albuminuria, the presence of excess of protein in urine, is a well-known risk factor for early kidney damage among diabetic/prediabetic patients. There is a complex interaction between physical activity (PA) and albuminuria. However, the relationship of specific-domain PA and albuminuria remained obscure.

**Methods:**

Albuminuria was defined as urinary albumin/creatinine ratio (ACR) > 30 mg/g. PA was self-reported by participants and classified into transportation-related PA (TPA), occupation-related PA (OPA), and leisure-time PA (LTPA). Weighted logistic regression was conducted to compute the odds ratios (ORs) and 95% confidence intervals (CIs). Restricted cubic spline (RCS) was used to evaluate the dose–response of PA domains with the risk of albuminuria.

**Results:**

A total of 6739 diabetic/prediabetic patients (mean age: 56.52 ± 0.29 years) were enrolled in our study, including 3181 (47.20%) females and 3558 (52.80%) males. Of them, 1578 (23.42%) were identified with albuminuria, and 5161(76.58%) were without albuminuria. Diabetic/prediabetic patients who adhered the PA guidelines for total PA had a 22% decreased risk of albuminuria (OR = 0.78, 95%CI 0.64–0.95), and those met the PA guidelines for LTPA had a 28% decreased of albuminuria (OR = 0.72, 95%CI 0.57–0.92). However, OPA and TPA were both not associated with decreased risk of albuminuria. RCS showed linear relationship between the risk of albuminuria with LTPA.

**Conclusions:**

Meeting the PA guideline for LTPA, but not OPA and TPA, was inversely related to the risk of albuminuria among diabetic/prediabetic patients. Additionally, achieving more than 300 min/week of LTPA conferred the positive effects in reducing albuminuria among diabetic/prediabetic patients.

**Supplementary Information:**

The online version contains supplementary material available at 10.1186/s12967-024-05061-6.

## Introduction

Diabetes mellitus (DM), a common and chronic metabolic disease, is characterized by prolonged elevated blood glucose and insulin resistance (IR), accompanied by complicated clinical complications [1]. Meanwhile, DM usually affects multiple organs involving the cerebrovascular, kidney, retina, peripheral nerves, and heart, which is the major reason for death and disability in patients with DM [2]. Timely detection and treatment of diabetes mediated organ damage can be effective in lessening the risk of fatal complications and healthcare expenditures [3]. Meanwhile, albuminuria has been regarded as a sensitive predictor of diabetic nephropathy (DN) [4]. It was a relatively early manifestation of kidney injury secondary to DM [5]. Besides, prediabetes refers to a blood glucose concentration that falls below the threshold for diabetes but exceeds the normal range. Of note, it is closely linked to an elevated risk of developing diabetes and its associated complications [6]. Indeed, albuminuria serves as a dependable comprehensive indicator for all the vascular complications linked to both prediabetes and diabetes [7]. A meta-analysis performed by Matsushita et al. revealed a positive association between albuminuria and all-cause mortality and cardiovascular mortality [8]. Furthermore, the results from a cohort study showed that decreased urinary albumin excretion was related to the decreased risk of cardiovascular events among patients with diabetes, emphasizing the significance of reducing the incidence of proteinuria in diabetes [9]. Therefore, effective early prevention, including developing a healthy lifestyle and diet, is urgently needed to prevent albuminuria among patients with diabetes and prediabetes.

To date, physical activity (PA) has been known to have countless profound benefits in enhancing physical condition by preventing diseases, for instance, cardiovascular disease, diabetes, renal dysfunction, and several cancers [10, 11]. In general, PA comprises three different domains, including occupation-related PA (OPA), transportation-related PA (TPA), and leisure-time PA (LTPA). Research based on the survey of the nutritional and health status of Korean discovered LTPA could reduce the risk of diabetes, while TPA was negatively related to diabetes incidence only in males, and OPA demonstrated no association with diabetes among Korean adults [12]. A previous clinical study involving 4661 adults showed that LTPA was beneficial to reduce the risk of diabetes, whereas domestic and work-related PA might potentially increase the risk [13]. Another research showed that LTPA, TPA, and OPA had similar health-enhancing effects on reducing diabetes risk among American adults [14]. Besides, the results from a meta-analysis demonstrated an inverse correlation between LTPA and OPA with the risk of diabetes [15]. However, another meta-analysis conducted by Raza et al. found that LTPA and TPA were related to a lower risk of DM [16]. Although numerous studies and meta-analysis examined the relationship between domain-specific PA and diabetes, all of them yielded different results. Previously published research demonstrated that higher levels of PA contributed to a reduction in urinary albumin excretion [17]. Until now, research on PA and albuminuria has generally centered on total PA, but few investigations have explored the relationship between three different PA domains and albuminuria risk. Elucidating the correlation between domain specificity and albuminuria risk is critical since PA varies across domains in terms of frequency, duration, and intensity.

Therefore, we investigated the underlying connection between domain-specific PA and albuminuria risk in patients with diabetes/prediabetes to help develop intervention and prevention strategies. The data used in our research was derived from National Health and Nutrition Examination Survey (NHANES) performed in the United States.

## Materials and methods

### Study design and population

NHANES, a periodic and nationally plan with a series of multistage stratified sample design, conducted by the National Center for Health Statistics (NCHS) to survey the nutritional and health status of US adults [18]. Besides, it’s not necessary to acquire additional ethical clearance because of the data's openness and originality. The NCHS signed all informed consent which was obtained from each participant at the time of recruitment. Detailed analytical procedures can be found at NHANES official website.

**Fig. 1:**
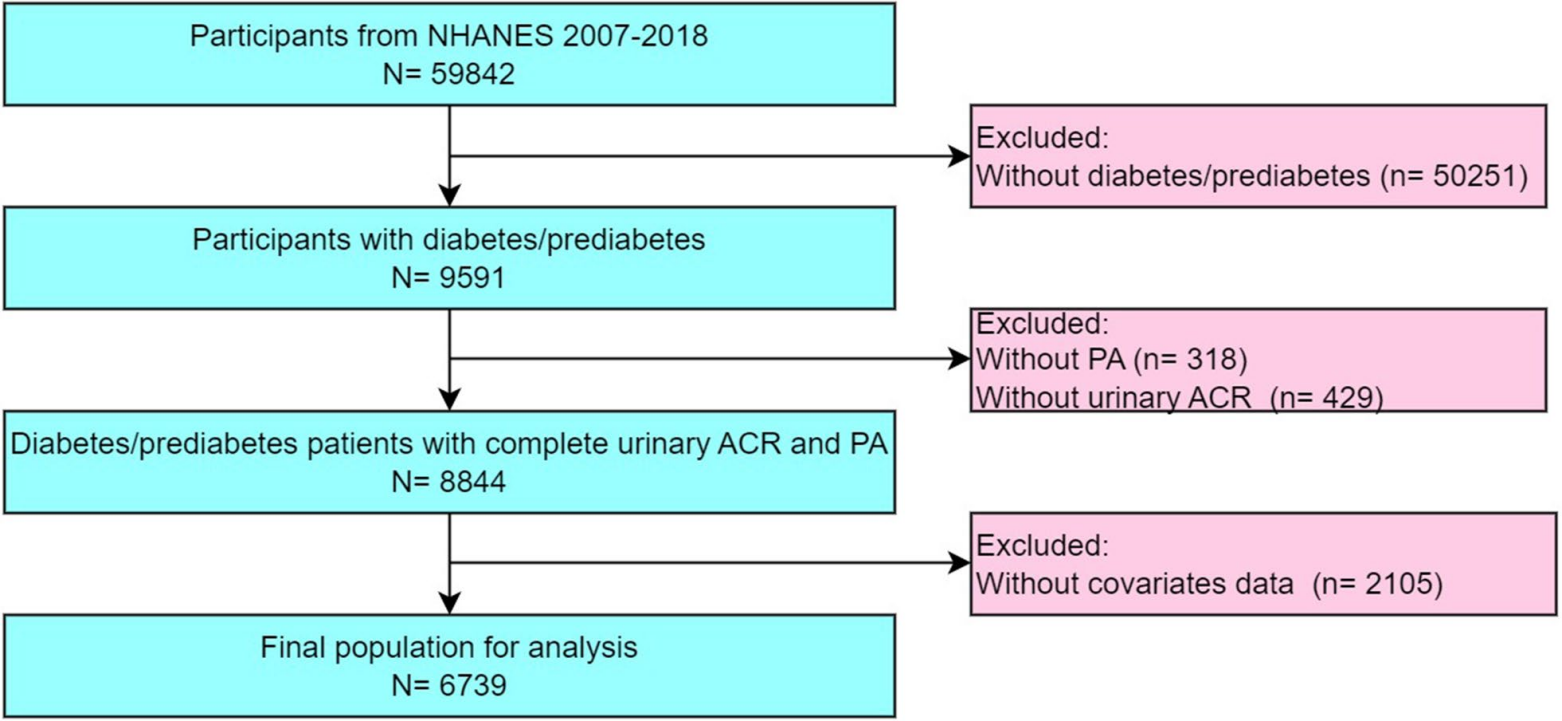
Flow diagram of the study design

The current study extracted public data from NHANES 2007–2018 to clarify the connection between PA domain and albuminuria risk among diabetic/prediabetic patients. Specifically, a total of 59,842 participants from these six continuous cycles were surveyed. Among them, only 9591 participants were defined as having diabetes/prediabetes. We further excluded participants with incomplete information of the following conditions: urinary albumin/creatinine ratio (ACR) (n = 429), PA (n = 318), and covariates data (n = 2105). Finally, we enrolled 6739 eligible diabetes/prediabetes patients in the current study (Fig. 1).

### Definition of PA

PA was evaluated by a series of self-reported PA questionnaires from NHANES [19], and all activities were divided into three parts: TPA, LTPA, and OPA. Obviously, the total PA consisted of TPA, OPA, and LTPA. At the same time, the duration (minute/week), frequency (time/week), and intensity (vigorous or moderate) of PA were recorded by the highly trained interviewer. In brief, vigorous/moderate PA was described as activities that required hard/moderate physical effort and caused a large/small increase in breathing or heart rate. Of note, 1 min of vigorous PA was equivalent to 2 min of moderate PA according to NHANES analytic guidelines [14].

The classification of PA was based on the 2018 PA guidelines, which suggest adults participate in a minimum of 150–300 min per week of PA with moderate intensity, 75–150 min per week of PA with vigorous intensity, or an equal combination of both [20]. Therefore, all participants were set into 2 categories: (1) participants who adhered to the 2018 PA guidelines (engaging in at least 150 min of PA in a typical week) and (2) participants who did not meet the 2018 PA guidelines (engaging in less than 150 min of PA in a typical week). Besides, to examine the dose–response effect of PA on albuminuria, we also divided these participants into 4 levels: (1) 0 min/week, (2) 1–149 min/week, (3) 150–299 min/week, and (4) ≥ 300 min/week [20, 21].

### Definition of diabetes/prediabetes

Participants with diabetes and prediabetes were considered as our study population. According to previous guidelines, individuals were classified as having diabetes if they satisfied any of the following conditions:(1) a physician confirmed a diabetes diagnosis; (2) the glycosylated hemoglobin (HbA1c) was above 6.5%; (3) fasting glucose was equal to or greater than 7.00 mmol/L; (4) random blood glucose level was equal to or greater than 11.10 mmol/L; (5) two-hour oral glucose tolerance test (OGTT) was equal to or greater than 11.10mmo/L; (6) current usage of any drugs or insulin for diabetes management. At the same time, prediabetes was classified as impaired fasting glycemia (IFG), which indicated fasting glucose levels ranging from 6.11 mmol/L to 7.00 mmol/L, and impaired glucose tolerance (IGT), which was characterized by a two-hour oral glucose tolerance test (OGTT) result between 7.70 mmol/L and 11.10 mmol/L [22].

### Definition of albuminuria

NHANES provided a series of urine sample data, and eligible participants were allowed to complete the measurement of urine in room mobile examination center (MEC). Urinary albumin and creatinine were measured by a solid-phase fluorescent immunoassay, a non-competitive, double-antibody method for determining human albumin in the urine. We divided the urinary albumin concentration (mg) by the urinary creatinine concentration (g) to obtain the urinary ACR, and then we defined ACR > 30 mg/g as albuminuria [23].

### Covariates

Our regressions models considered some potential confounders that have been correlated with albuminuria among diabetic/prediabetic patients: age, gender, race/ethnicity, education level, marital status, poverty income ratio (PIR), body mass index (BMI), smoking, alcohol status, cardiovascular disease (CVD), hypertension, serum creatinine, uric acid, hyperlipidemia, and medication use. Race/Ethnicity was categorized into Non-Hispanic Black, Non-Hispanic White, Mexican American, and other races. Marital status was classified into married, unmarried, and others. PIR was divided into 3 groups: ≤ 1.30, 1.30–3.50, and > 3.50. Education level was grouped into 5 levels: less than 9th grade, 9–11th grade, high school, some college, and college or above. Other covariates including smoking, alcohol status, CVD, and medication use were assessed by a set of self-reported questionnaires. According to a previous study, alcohol status was categorized as never (< 12 drinks in a lifetime), former (≥ 12 drinks in 1 year and did not drink last year), mild drinker (≤ 1 drink/day for women or ≤ 2 drinks/day for men), and heavy drinker (≥ 2 drinks/day for women or ≥ 3 drinks/day for men) [24]. The diagnosis for hypertension relied on one or more of the following three factors: (1) Participants affirmatively answered the blood pressure survey question "Have you ever been diagnosed with hypertension by a healthcare professional?" (2) Individuals were classified as hypertensive if their average systolic blood pressure (SBP) exceeded or equaled 130 mmHg or if their average diastolic blood pressure (DBP) exceeded or equaled 80 mmHg. (3) current usage of any drugs for hypertension management.[25]. Other biochemical parameters including uric acid, serum creatinine, and serum lipid levels were obtained from NHANES laboratory data.

### Statistical analysis

Based on the NHANES analysis recommendations, a complex sample design was fully taken into account, and appropriate sample weight was used to compute estimates in current study. P value less than 0.05 was regarded as significant, and all statistical tests were bilateral. R software (version 4.1.3) was carried out to analyze all data in current study.

Continuous variables were presented as mean and standard error (SE), and categorical variables were expressed as frequency with percentage. Chi-square test and Student’s t-test were carried out to assess individuals’ baseline characteristics according to the albuminuria status. A series of weighted logistic regressions were conducted to estimate odds ratios (ORs) and 95% confidence intervals (CIs) to analyze the connection between PA and albuminuria among diabetic/prediabetic patients. We established 3 statistical models to check our results in this study: model 1(a crude model); model 2 adjusted age, gender, and race/ethnicity; model 3 added to model 2, marital status, PIR, education level, BMI, smoking, alcohol status, CVD, hypertension, serum creatinine, uric acid, hyperlipidemia, and medication use. In addition, we transformed ACR with natural logarithm function (lnACR) to stabilize variance and then used multivariate linear regression to calculate the β value and 95%CI. Furthermore, we explored the association between PA and albuminuria risk among uncontrolled and controlled diabetic/prediabetic patients due to the fact that poorly controlled blood glucose could aggravate albuminuria. HbA1c ≥ 7.0 was defined as uncontrolled diabetes/prediabetes, and HbA1c < 7.0 was defined as controlled diabetes/prediabetes [26].To further visualize the association of PA and albuminuria, restricted cubic spline (RCS) was used to determine the dose–response. Subgroup analyses stratified by demographic features (age, gender, race/ethnicity, PIR, and marital status), BMI, and smoke were performed to examine whether the relationship was robust. Lastly, we used the likelihood ratio test to examine the interactions among these subgroups and nonlinearity in RCS.

Moreover, we further quantified the influence of total PA and LTPA on lipid metabolism, glucose metabolism, and inflammation. Lipid metabolism was assessed by four serum lipid markers, and glucose metabolism was evaluated by fast glucose and HbA1c. Additionally, we utilized the systemic immune-inflammation index (SII, platelets count × neutrophil/lymphocyte ratio) to reflect the local immune response and systemic inflammation, which was a good and promising indicator for predicting albuminuria risk [20].

## Results

### Baseline characteristics

As illustrated in Table 1, the sample size for 6-year NHANES cycles was 6739, including 5161 (76.58%) diabetic/prediabetic patients without albuminuria, 1578 (23.42%) diabetic/prediabetic patients with albuminuria. Of them, 3181 (47.20%) were females, and 3558 (52.80%) were males, with an average age was 56.52 ± 0.29 years old. Specifically, variables of age, race/ethnicity, education, marital status, PIR, serum creatinine, serum uric acid, hypertension, hyperlipidemia, CVD, alcohol status, and anti-diabetic drugs use were all associated with albuminuria (all *P* < 0.050). No significant differences between two groups were found in BMI, gender, smoke, and anti-hypertensive drugs ( all *P* > 0.050). Besides, 3365 (49.93%), 1941 (28.80%), 730 (10.83%), and 1600 (23.74%) diabetic/prediabetic patients achieved the recommendation of total PA, OPA, TPA, and LTPA, respectively.

**Table 1:** The baseline characteristics of study participants

Variables	Overall	Without albuminuria	With albuminuria	*P* value
Sample, n (%)	6739	5161(76.58)	1578(23.42)	
Age, years	56.52 ± 0.29	55.39 ± 0.33	61.15 ± 0.49	< 0.001
BMI, kg/m^2^	32.22 ± 0.14	32.11 ± 0.15	32.67 ± 0.32	0.109
Gender, n (%)				0.999
Female	3181(47.20)	2469(47.36)	712(47.37)	
Male	3558(52.80)	2692(52.64)	866(52.63)	
Race/Ethnicity, n (%)				< 0.001
Mexican Americans	1185(17.58)	882(8.86)	303(11.28)	
Non-Hispanic Black	1408(20.89)	1046(10.28)	362(13.47)	
Non-Hispanic White	2719(40.35)	2120(68.14)	599(61.79)	
Other races	1427(21.18)	1113(12.73)	314(13.46)	
Education, n (%)				< 0.001
Less Than 9th Grade	1004(14.90)	710(6.96)	294(12.17)	
9–11th Grade	1064(15.79)	785(11.64)	279(14.47)	
High School	1575(23.37)	1214(24.48)	361(26.46)	
Some College	1880(27.90)	1471(32.19)	409(28.40)	
College or above	1216(18.04)	981(24.73)	235(18.50)	
Marital status, n (%)				0.002
Married	3760(55.79)	2936(60.22)	824(55.54)	
Unmarried	704(10.45)	562(10.85)	142(9.47)	
Others	2275(33.76)	1663(28.93)	612(34.99)	
PIR, n (%)				< 0.001
≤ 1.30	2335(34.65)	1711(21.33)	624(30.30)	
1.30–3.50	2620(38.88)	1965(36.64)	655(40.43)	
> 3.50	1784(26.47)	1485(42.03)	299(29.27)	
Serum creatinine, μmol/L	82.19 ± 0.58	78.48 ± 0.39	97.36 ± 2.59	< 0.001
Serum uric acid, μmol/L	344.40 ± 1.55	341.27 ± 1.73	357.21 ± 3.21	< 0.001
Hypertension, n (%)	5036(74.73)	3659(69.39)	1377(86.84)	< 0.001
Hyperlipidemia, n (%)	5742(85.21)	4353(85.11)	1389(88.27)	0.031
Smoke, n (%)				0.011
Never	3434(50.95)	2694(51.25)	740(44.91)	
Former	2140(31.76)	1590(32.32)	550(37.76)	
Current	1165(17.29)	877(16.43)	288(17.33)	
CVD, n (%)	1348(20.00)	868(15.65)	480(27.91)	< 0.001
Alcohol status, n (%)				< 0.001
Never	1816(26.95)	1434(30.56)	382(25.44)	
Former	1510(22.41)	1076(17.22)	434(24.84)	
Mild	2264(33.60)	1793(39.23)	471(33.54)	
Heavy	1816(26.95)	1434(30.56)	382(25.44)	
Medication use, n (%)				
Anti-hypertensive drugs	822(12.20)	618(11.48)	204(12.28)	0.508
Anti-diabetic drugs	2844(42.20)	1937(34.31)	907(54.61)	< 0.001
Total PA: achieved, n (%)	3365(49.93)	2705(55.99)	660(42.84)	< 0.001
OPA: achieved, n (%)	1941(28.80)	1555(34.26)	386(27.25)	0.003
TPA: achieved, n (%)	730(10.83)	568(9.69)	162(8.27)	0.167
LTPA: achieved, n (%)	1600(23.74)	1329(28.73)	271(18.05)	< 0.001

Continuous variables were presented as mean ± SE, and categorical variables were presented as the frequency with percentage

BMI, body mass index; PIR, poverty income ratio; CVD, cardiovascular disease; PA, physical activity; OPA, occupation-related PA; TPA, transportation-related PA; LTPA, leisure-time PA; SE, standard error

### Association between PA and albuminuria

Results using multivariate logistic regression to analyze the association between PA and albuminuria are presented in Table 2. After controlling all potential covariates, we found that diabetic/prediabetic patients who met the PA guidelines for total PA had a 22% decreased risk of albuminuria (OR = 0.78, 95%CI 0.64–0.95), and those met the PA guidelines for LTPA had a 28% decreased of albuminuria (OR = 0.72, 95%CI 0.57–0.92). However, no significant associations of OPA and TPA with albuminuria were found among diabetic/prediabetic patients. The similar findings were discovered when we incorporated lnACR as a continuous factor within the same model.

**Table 2:** Association between PA domain and albuminuria among diabetic/prediabetic patients

	Model 1		Model 2		Model 3	
Categorical variable						
Albuminuria	OR (95%CI)	*P* value	OR (95%CI)	*P* value	OR (95%CI)	*P* value
Total PA						
No	Reference		Reference		Reference	
Yes	0.59 (0.50, 0.69)	< 0.001	0.66 (0.55, 0.79)	< 0.001	0.78 (0.64, 0.95)	0.014
Occupation-related PA						
No	Reference		Reference		Reference	
Yes	0.72 (0.58, 0.89)	0.003	0.82 (0.65, 1.03)	0.081	0.89 (0.70, 1.13)	0.327
Transportation-related PA						
No	Reference		Reference		Reference	
Yes	0.84 (0.66, 1.08)	0.168	0.89 (0.70, 1.13)	0.325	0.95 (0.74, 1.22)	0.690
Leisure-time PA						
No	Reference		Reference		Reference	
Yes	0.55 (0.44, 0.68)	< 0.001	0.60 (0.48, 0.74)	< 0.001	0.72 (0.56, 0.92)	0.009

	Model 1		Model 2		Model 3	
Continuous variable						
Ln ACR (mg/g)	β(95%CI)	*P* value	β(95%CI)	*P* value	β(95%CI)	*P* value
Total PA						
No	Reference		Reference		Reference	
Yes	− 0.15(− 0.19, − 0.11)	< 0.001	− 0.11(− 0.14, − 0.07)	< 0.001	− 0.06(− 0.10, − 0.03)	< 0.001
Occupation-related PA						
No	Reference		Reference		Reference	
Yes	− 0.09(− 0.13, − 0.05)	< 0.001	− 0.04(− 0.08, 0.00)	0.042	− 0.02(− 0.06, 0.01)	0.222
Transportation-related PA						
No	Reference		Reference		Reference	
Yes	− 0.05(− 0.11,0.00)	0.063	− 0.04(− 0.08, 0.01)	0.159	− 0.02(− 0.06, 0.03)	0.459
Leisure-time PA						
No	Reference		Reference		Reference	
Yes	− 0.15(− 0.19, − 0.12)	< 0.001	− 0.12(− 0.15, − 0.08)	< 0.001	− 0.07(− 0.11, − 0.03)	< 0.001

Model 1: None

Model 2: Age, gender, race/ethnicity

Model 3: Age, gender, race/ethnicity, marital status, PIR, education, BMI, smoking, alcohol status, CVD, hypertension, serum creatinine, uric acid, hyperlipidemia, anti-hypertensive drugs, and anti-diabetic drugs

ACR, Albumin/creatinine ratio; BMI, body mass index; PIR, poverty income ratio; CVD, cardiovascular disease; PA, physical activity; OPA, occupation-related PA; TPA, transportation-related PA; LTPA, leisure-time PA

As illustrated in Table 3, we further explored PA and albuminuria associations among patients with controlled and uncontrolled diabetes/prediabetes. For the controlled diabetes/prediabetes group, patients who met the PA guidelines for total PA had a 28% lower risk of albuminuria (OR = 0.72, 95%CI 0.56–0.92) and patients who met the PA guidelines for LTPA had a 31% lower risk of albuminuria (OR = 0.69, 95%CI 0.50–0.95) in the fully adjusted model. However, the associations of total PA and three different PA with albuminuria were not observed among patients with uncontrolled diabetes/prediabetes. Similar results were observed when we transformed albuminuria into continuous variable (Additional file 1: Table S1).

**Table 3:** Associations of PA domain and albuminuria in patients with controlled and uncontrolled blood glucose

	Controlled diabetes/prediabetes (n = 4851)					
	Model 1		Model 2		Model 3	
	OR (95%CI)	*P* value	OR (95%CI)	*P* value	OR (95%CI)	*P* value
Total PA						
No	Reference	Reference	Reference	Reference	Reference	Reference
Yes	0.53 (0.44, 0.65)	< 0.001	0.61 (0.49, 0.76)	< 0.001	0.72 (0.56, 0.92)	0.009
Occupation-related PA						
No	Reference	Reference	Reference	Reference	Reference	Reference
Yes	0.66 (0.50, 0.87)	0.003	0.76 (0.56, 1.02)	0.063	0.83 (0.61, 1.13)	0.223
Transportation-related PA						
No	Reference	Reference	Reference	Reference	Reference	Reference
Yes	0.72 (0.53, 0.98)	0.037	0.78 (0.57, 1.07)	0.119	0.83 (0.60, 1.15)	0.247
Leisure-time PA						
No	Reference	Reference	Reference	Reference	Reference	Reference
Yes	0.52 (0.39, 0.68)	< 0.001	0.58 (0.44, 0.77)	< 0.001	0.69 (0.49, 0.95)	0.024

	Uncontrolled diabetes/prediabetes (n = 1888)					
	Model 1		Model 2		Model 3	
	OR (95%CI)	*P* value	OR (95%CI)	*P* value	OR (95%CI)	*P* value
Total PA						
No	Reference	Reference	Reference	Reference	Reference	Reference
Yes	0.78(0.58,1.05)	0.102	0.83(0.61,1.14)	0.244	0.90(0.65,1.25)	0.534
Occupation-related PA						
No	Reference	Reference	Reference	Reference	Reference	Reference
Yes	0.87(0.60,1.25)	0.444	0.96(0.65,1.40)	0.812	0.97(0.66,1.43)	0.870
Transportation-related PA						
No	Reference	Reference	Reference	Reference	Reference	Reference
Yes	1.10(0.75,1.62)	0.631	1.13(0.78,1.65)	0.518	1.24(0.82,1.88)	0.295
Leisure-time PA						
No	Reference	Reference	Reference	Reference	Reference	Reference
Yes	0.70(0.49,1.01)	0.054	0.73(0.50,1.05)	0.090	0.80(0.56,1.15)	0.217

Model 1: None

Model 2: Age, gender, race/ethnicity

Model 3: Age, gender, race/ethnicity, marital status, PIR, education, BMI, smoking, alcohol status, CVD, hypertension, serum creatinine, uric acid, hyperlipidemia, anti-hypertensive drugs, and anti-diabetic drugs

BMI, body mass index; PIR, poverty income ratio; CVD, cardiovascular disease; PA, physical activity; OPA, occupation-related PA; TPA, transportation-related PA; LTPA, leisure-time PA

**Fig. 2:**
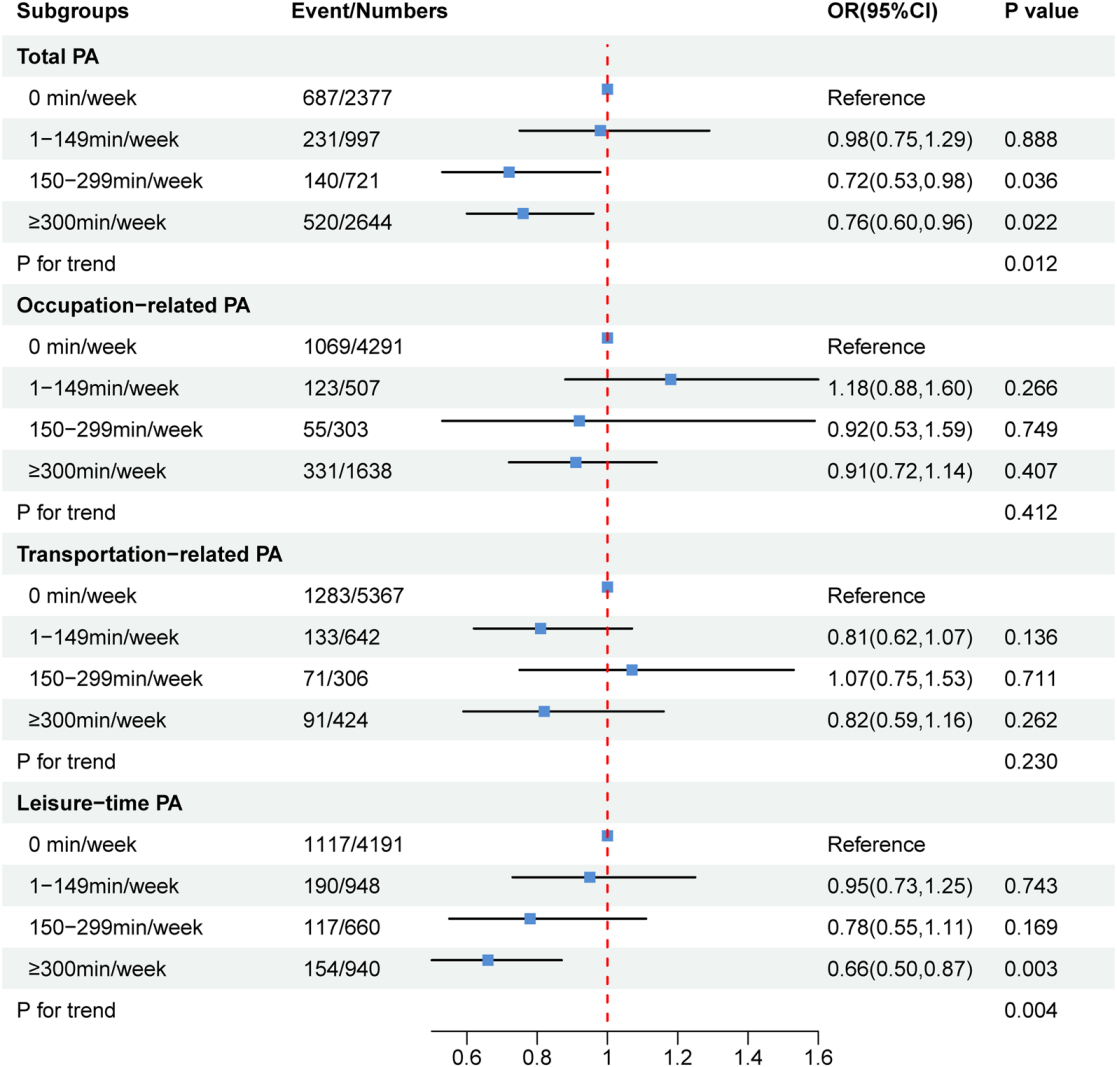
Multivariable OR for albuminuria among diabetic/prediabetic patients based on the amount of PA

**Fig. 3:**
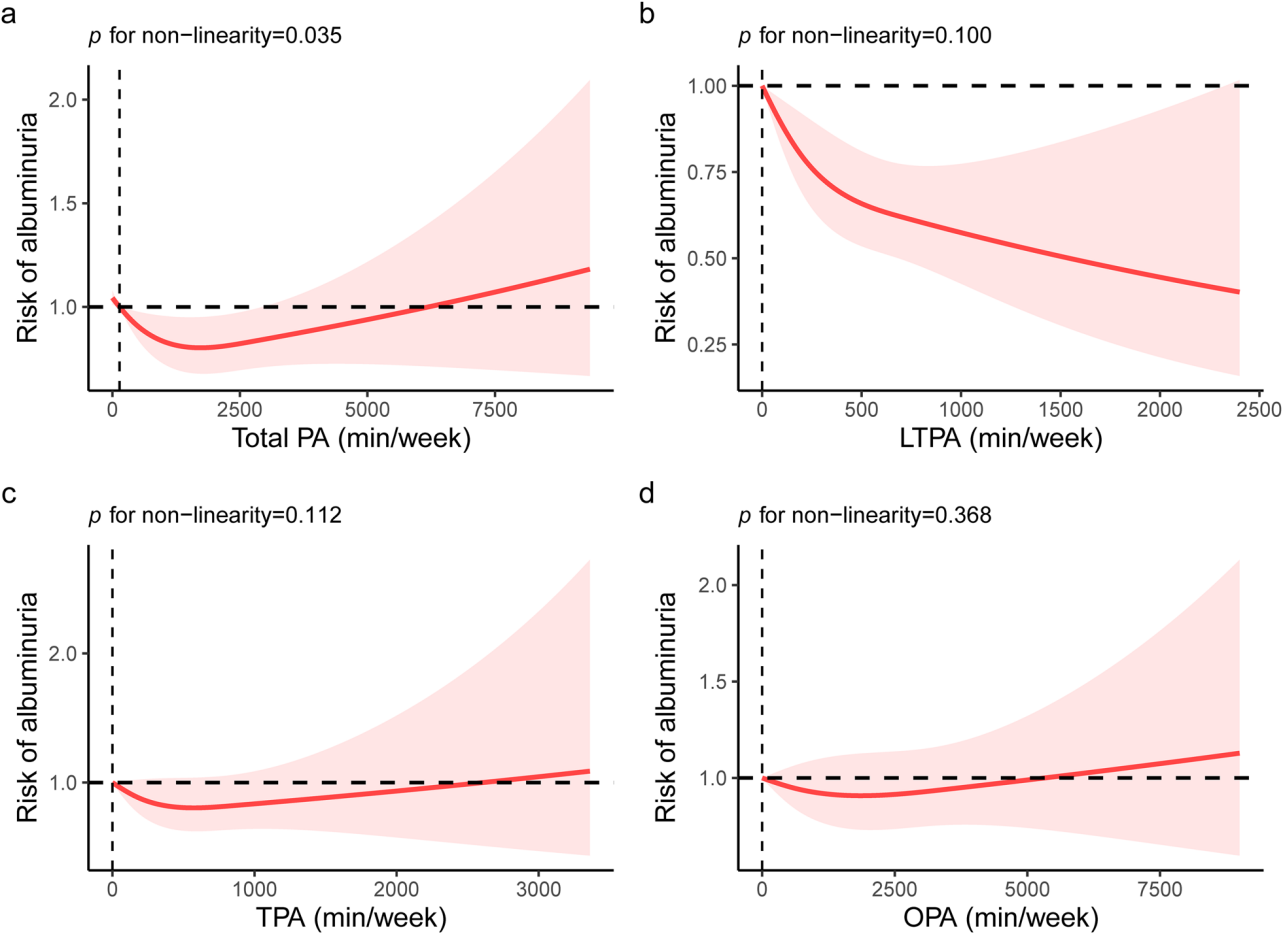
Restricted cubic spline analysis (RCS) with multivariate-adjusted associations between PA domains and the risk of albuminuria among diabetic/prediabetic patients. **a** RCS analysis between total PA and albuminuria risk. **b** RCS analysis between LTPA and albuminuria risk. **c** RCS analysis between TPA and albuminuria risk. **d** RCS analysis between OPA and albuminuria risk

Furthermore, in order to examine the dose–response relationship of domain-specific PA and albuminuria risk, we stratified the amount of PA into 4 levels: 0 min/week, 1–149 min/week, 150–299 min/week, and ≥ 300 min/week (Fig. 2). Interestingly, we also found higher levels of total PA and LTPA were both inversely associated with albuminuria among diabetic/prediabetic patients. Specifically, for LTPA, diabetic/prediabetic patients who completed ≥ 300 min/week had 34% (OR = 0.66, 95%CI 0.50–0.87) decreased risk of albuminuria. Nevertheless, OPA and TPA were still not linked to the risk of albuminuria (both *P* > 0.050). Additionally, multivariable adjusted RCS analysis showed the non-linear relationship between the risk of albuminuria with total PA (*P* for non-linearity = 0.033, Fig. 3a), We also observed the linear relationship of LTPA, TPA, and OPA with the risk of albuminuria among diabetic/prediabetic patients (all *P* for non-linearity > 0.050, Fig. 3b, c, d).

Lastly, we investigated the relationship between each PA domain and glucose metabolism, lipid metabolism, and inflammation. Compared with those who completed less than 150 min/week of total PA and LTPA, diabetic/prediabetic patients who completed more than 150 min/week had higher high-density lipoprotein (HDL) cholesterol, and lower glucose, HbA1c, and SII (Additional file 1: Table S2).

### Subgroup analysis

**Fig. 4:**
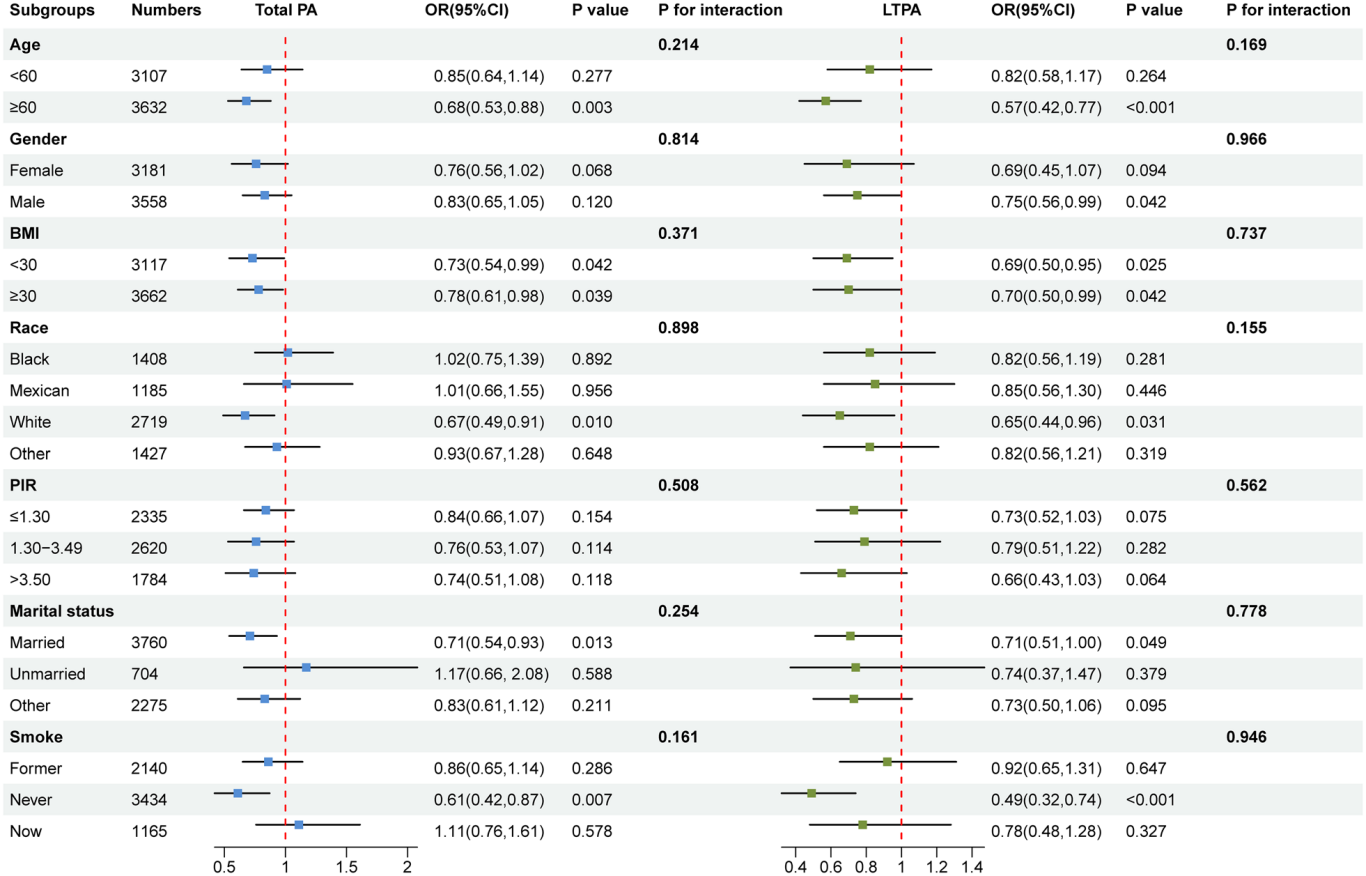
Subgroup analysis of the association of total PA and LTPA with albuminuria risk among diabetic/prediabetic patients

Subgroup analyses of the associations of total PA and LTPA with albuminuria among diabetic/prediabetic patients are shown in Fig. 4. Among most subgroups, the associations of total PA and LTPA with reduced albuminuria among diabetic/prediabetic patients were consistent. However, no significant interaction of each stratum with total PA and LTPA with albuminuria was found (all *P* for interaction > 0.050).

## Discussion

In this large cross-sectional evidence from NHANES, we discovered that meeting the PA guideline for total PA and LTPA was inversely related to the risk of albuminuria among diabetic/prediabetic patients. More importantly, achieving more than 300 min/week of LTPA conferred the positive effects in reducing albuminuria among these patients. However, the associations of OPA and TPA with reduced risk of albuminuria were not found. These findings highlighted the beneficial effect of LTPA on albuminuria, suggesting a modifiable lifestyle for diabetic/prediabetic patients to prevent albuminuria was an effective and promising strategy.

Currently, the majority of research has focused on the correlation between total PA and albuminuria incidents, while little research has explored if the same health-enhancing effects can be found in different domain-specific PA. The protective effect of renal function on routine physical training in diabetes rats has been reported by previous basic research [27]. Another clinical research has observed an opposed association between PA and urinary albumin excretion in 3,587 participants, indicating that adherence to active exercise could have profound benefits in preventing albuminuria [17]. Similarly, the results from recent observational research performed by Böhm et al. suggested that regular PA was inversely related to the new onset of albuminuria in 31,312 subjects, further showing that active exercise can confer benefits on decreasing albuminuria risk [28]. A meta-analysis used a continuous metric for PA to explore the effect of PA on type 2 diabetes incidence, indicating that PA had profound benefits in preventing diabetes [29]. Another research emphasized lifestyle intervention, such as active exercise, played a key role in the management of diabetic patients [30]. A prospective study enrolled 1,390 participants and found that LTPA, especially of high intensity, performed a protective role against development of DN in type 1 diabetes [31]. The impacts of different PA domains in preventing DM and improving prognosis were well established in previous investigations [12–16]. The above studies directly or indirectly supported our results in current research. In another study conducted by Robinson et al., it was similarly discovered that engaging in high levels of PA, specifically including walking and strenuous sports, was associated with a lower risk of albuminuria in 2 US cohorts [17]. Although the consistent findings between their study and ours were observed, it was noteworthy that they only focused on the nondiabetic women and ignored investigating the dose-relationship of TPA and OPA with albuminuria risk. Besides, 40.35% Non-Hispanic White, 20.89% Non-Hispanic Black, and 17.58% Mexican Americans were included in our study, whereas theirs consisted of 96% Non-Hispanic White participants. Data from NHANES III indicated that US racial/ethnic minority groups with and without diabetes had greater occurrence of albuminuria compared to non-Hispanic whites without diabetes. Therefore, race/ethnicity should be considered as a risk factor and to adjust [32]. Endothelial dysfunction is often accompanied by diabetic patients, which is also thought to be one of the causes of kidney injury secondary to DM [33]. It is well known that regular PA could preserve endothelial function via several mechanisms, such as promoting antioxidant effects, increasing nitric oxide production and its bioavailability [34]. Two previous meta-analysis assessed the relationship between exercise interventions and endothelial function measured by brachial artery flow-mediated dilation, demonstrating the benefits of exercise on endothelial function in diabetic patients, especially aerobic and combined exercise [35, 36].

We speculated that active physical exercise could protect renal function by enhancing microvascular endothelial function. In addition, we also found a dose–response relationship between LTPA and albuminuria events, proving more exercise during leisure time is more beneficial. Therefore, individuals with diabetes/prediabetes, especially those who lack exercise, should pay more attention to aerobic exercise to prevent the onset of albuminuria.

As we all know, prolonged hyperglycemia, hyperinsulinemia, chronic inflammation, and lipid metabolism disorder in diabetic/prediabetic patients can result in renal injury early manifested as albuminuria [5, 37]**.** A novel finding in our analysis was that LTPA based on meeting PA guidelines, but not OPA or TPA could reduce system inflammation and improve insulin resistance and lipid metabolism among diabetic/prediabetic patients. In addition, we only found a negative correlation between LTPA and albuminuria events in the controlled HbA1c groups, demonstrating the central role of glycemic control in reducing the risk of albuminuria. The results from a longitudinal cohort study documented that elevated fasting plasma glucose was recognized as a significant and independent risk factor for albuminuria events [38]. Insulin resistance can affect renal hemodynamics and cause glomerular dysfunction and glomerulosclerosis, ultimately leading to albuminuria [39, 40]. Besides, Mone et al. suggested that the management of IR could be taken seriously to reduce the risk of complications [41]. Moreover, excessive inflammatory cell factors can directly impair renal glomerular function and correlate with an increased urinary protein excretion rate [42]. Indeed, endothelial dysfunction, inflammation, and oxidative stress are often accompanied by diabetic patients, all of which are risk factors of albuminuria [43]. Several studies suggested targeting lipid metabolism disorders may be a key strategy for the treatment of proteinuric renal disease [44]. Thus, we speculated that adherence to LTPA, but not OPA or TPA, could decrease the urinary protein excretion rate by counteracting the harmful effects of hyperglycemia, insulin resistance, inflammation and lipid dysmetabolism on renal function. Furthermore, a recent cohort study conducted by Yen and his colleagues exhibited a significant negative correlation between LTPA and risk of all-cause and cardiovascular death in 4859 adults with diabetes [45]**.** On the whole, adherence to PA in daily life, especially during leisure time, was of crucial significance for the management of diabetic/prediabetic patients.

We did not observe beneficial impacts of OPA and TPA on reducing albuminuria risk in diabetic/prediabetic patients. Consistent with our findings, several studies have suggested OPA and TPA can not offer the same health benefits as LTPA. A prospective cohort study conducted by Lund et al. found that physical exposure in the work environment was associated with prolonged sickness absence, which impaired physical health to some extent [46]. Treff et al. indicated that individuals with more activity during transportation could be greater exposure to traffic air pollution [47]. Mengozzi and his colleagues observed that excessive exposure to diethylhexyl phthalate metabolites was associated with albuminuria incidents in diabetic patients [48]. Another study found that particulate matter 2.5 exposure was harmful to renal function, increasing the risk of albuminuria and CKD [49]. Additionally, the result from a cross-sectional study found a positive association between polycyclic aromatic hydrocarbons exposure and ACR levels [50]. Besides, another speculation to explain the phenomenon was that the intensity of OPA and TPA by participants in our study may not be sufficient to provide a prophylactic benefit against albuminuria. Taken together, we hold that diabetic/prediabetic patients can not derive benefits from OPA and TPA. Further prospective studies are needed to confirm our conjecture.

Our research has several strengths to be noted. First, our research data is established on the NHANES database, a large-scale national investigation, which helps us ensure the reliability of our results. Second, the ACR is an accurate and sensitive index with fewer influencing factors, relatively stable in individuals, enabling a better assessment of early renal damage in patients with diabetes/prediabetes. Third, stratified analyses and interaction analyses were performed to improve the stability of our findings.

Of note, several shortcomings should be pointed out in our research. First, causality can not be established owing to the restrictions of the cross-sectional design. Hence, necessitating additional prospective studies to corroborate our findings. Second, our findings should be cautiously interpreted when extending to additional regions or countries since only Americans with diabetes/prediabetes were enrolled in our study. Third, our study focused on different PA domains without considering the influence of time of day on PA because a recent report suggested that participants who exercised in midday-afternoon (11:00–17:00) had a lower risk of CVD and all-cause mortality [51]. Fourth, PA was assessed through self-reported survey questionnaires rather than employing objective measurement methods such as accelerometers. Nevertheless, the PA questionnaires were more suitable for our study, offering a superior evaluation of domain-specific PA compared to device-based measurements. Fifth, owing to the limitations of the NHANES database, we lacked information on the specific classification of DM. However, given the age of subjects enrolled in current study, we speculated that the majority likely have type 2 diabetes. More prospective studies are needed to confirm the relationship between PA domains and albuminuria risk among different types of diabetes. Finally, whereas common confounding factors were fully considered in the present investigation, other unknown and unmeasured confounding factors were not controlled.

## Conclusion

In summary, meeting the PA guideline for LTPA, but not OPA and TPA, was inversely related to the risk of albuminuria among diabetic/prediabetic patients. Additionally, achieving more than 300 min/week of LTPA conferred the positive effects in reducing albuminuria among these patients. More well-designed and longitudinal studies are urgent to validate our findings.

### Supplementary Information

**Additional file 1:** **Table S1.** Associations of PA domain and albuminuria in patients with controlled and uncontrolled blood glucose. **Table S2.** Domain-specific PA and glucose metabolism, lipid metabolism, and inflammation.

## Data Availability

Data described in the manuscript are publicly and freely available without. restriction at https://www.cdc.gov/nchs/nhanes/index.htm.

## Abbreviations

ACR: Albumin/creatinine ratio
BMI: Body mass index
CI: Confidence interval
CVD: Cardiovascular disease
DM: Diabetes mellitus
DN: Diabetic nephropathy
HbA1c: Glycosylated hemoglobin
HDL: High-density lipoprotein
IFG: Impaired fasting glycemia
IGT: Impaired glucose tolerance
IR: Insulin resistance
LDL: Low-density lipoprotein
lnACR: ACR with natural logarithm function
LTPA: Leisure-time physical activity
NCHS: National Center for Health Statistics
NHANES: National Health and Nutrition Examination Survey
OGTT: Oral glucose tolerance test
OPA: Occupation-related physical activity
OR: Odds ratio
PA: Physical activity
PIR: Poverty income ratio
RCS: Restricted cubic spline
SE: Standard error
SII: Systemic immune-inflammation index
TPA: Transportation-related physical activity
