# Supplementary material for: Association of domain-specific physical activity with albuminuria among prediabetes and diabetes: a large cross-sectional study
Source: J Transl Med. 2024 Mar 8;22:252. doi: 10.1186/s12967-024-05061-6 (PMC10921818; doi:10.1186/s12967-024-05061-6)
Supplement: Supplementary file 1 — Additional file 1: Table S1. Associations of PA domain and albuminuria in patients with controlled and uncontrolled blood glucose. Table S2. Domain-specific PA and glucose metabolism, lipid metabolism, and inflammation. [file 12967_2024_5061_MOESM1_ESM.docx]

Additional file 1: Table S1. Associations of PA domain and albuminuria in patients with controlled and uncontrolled blood glucose.

|  | **Controlled diabetes/prediabetes (n= 4851)** | | | | | |
| --- | --- | --- | --- | --- | --- | --- |
|  | Model 1 |  | Model 2 |  | Model 3 |  |
|  | β(95%CI) | *P* value | β(95%CI) | *P* value | β(95%CI) | *P* value |
| **Total PA** |  |  |  |  |  |  |
| No | Reference | Reference | Reference | Reference | Reference | Reference |
| Yes | -0.15(-0.19,-0.11) | <0.001 | -0.10(-0.14,-0.06) | <0.001 | -0.07(-0.11,-0.03) | 0.001 |
| **Occupation-related PA** |  |  |  |  |  |  |
| No | Reference | Reference | Reference | Reference | Reference | Reference |
| Yes | -0.09(-0.14,-0.05) | <0.001 | -0.05(-0.09, 0.00) | 0.045 | -0.03(-0.08, 0.01) | 0.153 |
| **Transportation-related PA** |  |  |  |  |  |  |
| No | Reference | Reference | Reference | Reference | Reference | Reference |
| Yes | -0.07(-0.12,-0.03) | 0.002 | -0.05(-0.09,-0.01) | 0.026 | -0.04(-0.08, 0.01) | 0.066 |
| **Leisure-time PA** |  |  |  |  |  |  |
| No | Reference | Reference | Reference | Reference | Reference | Reference |
| Yes | -0.13(-0.17,-0.09) | <0.001 | -0.09(-0.13,-0.05) | <0.001 | -0.05(-0.09,-0.01) | 0.016 |
|  | **Uncontrolled diabetes/prediabetes (n= 1888)** | | | | | |
|  | Model 1 |  | Model 2 |  | Model 3 |  |
|  | β(95%CI) | *P* value | β(95%CI) | *P* value | β(95%CI) | *P* value |
| **Total PA** |  |  |  |  |  |  |
| No | Reference | Reference | Reference | Reference | Reference | Reference |
| Yes | -0.08(-0.18,0.02) | 0.121 | -0.07(-0.17, 0.03) | 0.168 | -0.03(-0.12,0.06) | 0.520 |
| **Occupation-related PA** |  |  |  |  |  |  |
| No | Reference | Reference | Reference | Reference | Reference | Reference |
| Yes | -0.04(-0.15,0.07) | 0.501 | -0.01(-0.12, 0.10) | 0.830 | -0.01(-0.10,0.09) | 0.916 |
| **Transportation-related PA** |  |  |  |  |  |  |
| No | Reference | Reference | Reference | Reference | Reference | Reference |
| Yes | 0.03(-0.08,0.14) | 0.545 | 0.03(-0.08, 0.13) | 0.641 | 0.05(-0.06, 0.16) | 0.365 |
| **Leisure-time PA** |  |  |  |  |  |  |
| No | Reference | Reference | Reference | Reference | Reference | Reference |
| Yes | -0.15(-0.26,-0.04) | 0.007 | -0.15(-0.25,-0.04) | 0.007 | -0.09(-0.19, 0.02) | 0.103 |

Model 1: None

Model 2: Age, gender, race/ethnicity

Model 3: Age, gender, race/ethnicity, marital status, PIR, education, BMI, smoking, alcohol status, CVD, hypertension, serum creatinine, uric acid, hyperlipidemia, anti-hypertensive drugs, and anti-diabetic drugs.

Additional file 1: Table S2. Domain-specific PA and glucose metabolism, lipid metabolism, and inflammation*.

|  | **Total PA** | | **Leisure-time PA** | |
| --- | --- | --- | --- | --- |
|  | <150 min/week | ≥ 150 min/week | < 150 min/week | ≥ 150 min/week |
| **Glucose metabolism^a^** |  |  |  |  |
| Fast glucose | Reference | -0.23(-0.43,-0.02),0.029 | Reference | -0.26(-0.50,-0.02),0.032 |
| HbA1c | Reference | -0.09(-0.18, -0.01),0.038 | Reference | -0.11(-0.21,-0.01),0.031 |
| **Lipid metabolism^b^** |  |  |  |  |
| Total cholesterol | Reference | 0.04(-0.03, 0.12),0.277 | Reference | 0.01(-0.09, 0.10),0.906 |
| Triglyceride | Reference | -0.04(-0.13,0.05),0.348 | Reference | -0.09(-0.23, 0.06),0.233 |
| HDL cholesterol | Reference | 0.00(-0.02, 0.02),0.694 | Reference | 0.05(0.02, 0.07),0.002 |
| LDL cholesterol | Reference | 0.09( 0.00, 0.18),0.057 | Reference | -0.01(-0.10, 0.11),0.939 |
| **Inflammation^c^** |  |  |  |  |
| SII | Reference | -37.90(-61.67,-14.13),0.002 | Reference | -42.33(-66.61,-18.05),<0.001 |

* Value are expressed as β with 95% confidence intervals (CIs), and *p* value.

^a^ Adjusted for age, gender, race/ethnicity, marital status, PIR, education, BMI, smoking, alcohol status, CVD, hypertension, serum creatinine, uric acid, hyperlipidemia, and anti-hypertensive drugs. Of note, anti-diabetic drugs were not adjusted.

^b^Adjusted for age, gender, race/ethnicity, marital status, PIR, education, BMI, smoking, alcohol status, CVD, hypertension, serum creatinine, uric acid, anti-hypertensive drugs, and anti-diabetic drugs. Of note, hyperlipidemia was not adjusted.

^c^ Adjusted for age, gender, race/ethnicity, marital status, PIR, education, BMI, smoking, alcohol status, CVD, hypertension, serum creatinine, uric acid, hyperlipidemia, anti-hypertensive drugs, and anti-diabetic drugs.
